# Preparation of Polypropylene Micro and Nanofibers by Electrostatic-Assisted Melt Blown and Their Application

**DOI:** 10.3390/polym10090959

**Published:** 2018-08-29

**Authors:** Yi Pu, Jie Zheng, Fuxing Chen, Yunze Long, Han Wu, Qiusheng Li, Shuxin Yu, Xiaoxiong Wang, Xin Ning

**Affiliations:** 1 Industrial Research Institute of Nonwovens & Technical Textiles, College of Textiles &Clothing, Qingdao University, Qingdao 266071, China; puyiconan@163.com (Y.P.); fuxing1991@gmail.com (F.C.); wu71642@163.com (H.W.); lqs95007@163.com (Q.L.); 2 Collaborative Innovation Center for Nanomaterials & Devices, College of Physics, Qingdao University, Qingdao 266071, China; yunze.long@163.com (Y.L.); yushuxin326@163.com (S.Y.); wangxiaoxiong69@163.com (X.W.)

**Keywords:** electrostatic-assisted melt blown, microfibers, filtration efficiency

## Abstract

In this paper, a novel electrostatic-assisted melt blown process was reported to produce polypropylene (PP) microfibers with a diameter as fine as 600 nm. The morphology, web structure, pore size distribution, filtration efficiency, and the stress and strain behavior of the PP nonwoven fabric thus prepared were characterized. By introducing an electrostatic field into the conventional melt-blown apparatus, the average diameter of the melt-blown fibers was reduced from 1.69 to 0.96 μm with the experimental setup, and the distribution of fiber diameters was narrower, which resulted in a filter medium with smaller average pore size and improved filtration efficiency. The polymer microfibers prepared by this electrostatic-assisted melt blown method may be adapted in a continuous melt blown process for the production of filtration media used in air filters, dust masks, and so on.

## 1. Introduction

Nonwoven fabrics are a wide range of fibrous materials formed through direct fiber web formation rather than through yarn spinning and weaving. The fiber web is then bonded together by physical entanglement, thermal-, or chemical-bonding technologies [1]. Melt blown (MB) is one of the commercial nonwoven technologies whereby fine fibers (1–8 microns typically) are obtained in a single process of polymer fiber spinning, air quenching/drawing, and web formation [2]. During melt blown process, the polymer is fed into an extruder, where the polymer is melted and pushed forward by the extruder through the filter and ultimately reaches the spinning head. At the spinning head, the melt is drawn into filaments by high-speed hot air and forms nonwovens on the webformer [3,4]. Melt-blown nonwovens typically have fiber diameters ranging from 1 to 10 μm and the average diameter is generally 1–2 μm [4]. Melt blown has high production efficiency compared with other fine-fiber forming techniques. It can be several orders of magnitude higher in productivity than electrospinning, for example. Melt-blown fabric is known for its high surface area per unit weight and high barrier properties [4,5,6]. To improve the filtration efficiency, it is always desirable to further reduce fiber diameters, but there is a technology limit on the air speed and air volume which can be applied to the process, as the energy and equipment design requirements become economically unfeasible [5,7,8].

In the commercial interest of ultra-fine melt-blown nonwovens, many techniques have been attempted to make finer fibers. In 2009, a group used ultrasonic waves in melt blown, and the diameter of the prepared PET/PA6 bicomponent melt-blown webs was reduced from 3.62 to 2.11 μm [7]. In 2013, new die configurations and process conditions were explored to reduce the fiber size to the range of 300–500 nm [9]. In addition, some new technical methods have been developed in recent years to improve the performance and application value of melt-blown fibers [10,11,12,13,14]. In this paper, we explored the idea of adding a static electrical field to the melt blown process. In doing this, we reference electrospinning, which is a simple method of producing nanofibers under electrostatic force. It has largely been confined to the use of solution electrospinning as polymer melt-electrospinning is hindered by the very high polymer viscosity in the melt [15,16]. If we could combine the benefits of the two techniques in a way so that their shortcomings are mitigated, then we may be able to achieve a fibrous web with finer fiber diameters at a high production rate.

There have been some similar studies in this area. The idea of combining melt blowing and electrospinning was first proposed by Moosmayer et al. [17] in 1990. The concept was later incorporated in a technique termed “electroblowing”, by which a charged polymer melt is extruded through a spinneret to form nanofibers under the dual action of a high-velocity hot air stream and electric field [18]. The process extrudes electrically charged polymeric fluid through a spinneret, which is coupled to an air stream forwarding in the same direction as the extruding spinline. Together, both air stream and the electrostatic forces act on the spinline and fine fibers are obtained [19]. In 2009, a multihead melt-blowing electrospinning machine was developed by Watanabe et al. [20] The machine can use air blowing force and electrostatic force synergistically to prepare nanofibers. They successfully prepared nonwoven isotactic polypropylene fibers by this machine. In 2013, polypropylene fiber was prepared by a needleless melt-electrospinning device for marine oil-spill cleanup [16]. In 2017, Chen et al. [5] prepared nanofibers using melt electroblowing spinning, and the effects of air velocity and air temperature on fiber diameter were studied in detail. In 2017, Meng et al. [6] conducted a numerical simulation of the electrostatic field of electrospinning and the air flow field of melt blowing and discussed the combination of electrostatic force and air blowing force. They concluded that the combination of static electric force and air drawing force may be a good solution to produce nanofibers from a high-viscosity melt. The current literature mostly takes electrospinning as the starting point and the primary driver for obtaining fine fibers. In doing so, the commercial prospect of those techniques has never been truly verified, as the productivity has always been very low.

In recent years, with more and more attention paid to the living environment and human health, air filtration technology and products have been a major application area of technical textiles [21]. For a conventional filter material, particles are captured by sieving, inertial impact, and diffusion [22], depending on the size of the particles being removed. The probability of particle deposition on microfibers is greatly enhanced at smaller fiber diameters and higher surface areas [23,24]. Furthermore, the smaller particles tend to be trapped through the diffusion mechanism, and they have a greater chance of being adsorbed onto the charged surfaces of fibers [25].

The most commonly used method for improving the filtration efficiency of given fibrous structures is the static electret discharge process, by which light and small particles will be attracted toward the corona-charged fibers [26,27,28]. Different from the lower voltage, low action distance, and short operating time in the discharge process of static electret, the current electrostatic-assisted melt-blown process explores a larger voltage and longer action distance and operating time. Furthermore, the discharge process of static electret works only on solidified nonwoven fibers, while the electrostatic field of the present method works on both the melt and the solidified fibers, which provides an additional stretching for the charged fibers during the process of fiber attenuation. The filtration efficiency of nonwoven filter media obtained by electrostatic-assisted melt blown was shown in the present study to be much improved under the combined actions of the above two aspects.

In this study, the common melt-blown nonwoven system/equipment was modified, and an electrostatic field was directly applied adjacent to the melt-blown head to achieve a combination of melt blown and electric field effect, namely electrostatic-assisted melt blown. It is different from electroblowing in the electrospinning literature in which the melt stream is directly connected to an electrode for charged melt streams. Electrostatic-assisted melt blown is based on a commercial process without altering or charging the melt stream before spinning, therefore preserving the productivity attributes of a regular MB. The external charging device is to impose an electric field effect on the extruded melt stream after it is airborne. Polypropylene microfibers prepared in this way showed smaller fiber diameter and more concentrated size distribution. The effect of electric field intensity on fiber fineness and performance differences between electrostatic-assisted melt-blown fabrics and conventional melt-blown fabrics regarding fabric strength, pore size distribution, and filtration efficiency were studied in detail.

## 2. Experimental

### 2.1. Materials

Polypropylene (PP) was supplied from Shandong Dawn Polymer Material Co., Ltd. (Yantai, China). The product code is Z-1500, the melt flow rate (MFR) is 1500 ± 100 (tested under the GB/T 3682-2000 standard [29]), the nominal molecular weight is around 80,000, the ash content is ≤200 PPM (tested under the GB/T 9345.1-2008 standard [30]), and the moisture content is ≤0.2%.

### 2.2. Electrostatic-Assisted Melt-Blown Setup

**Figure 1:**
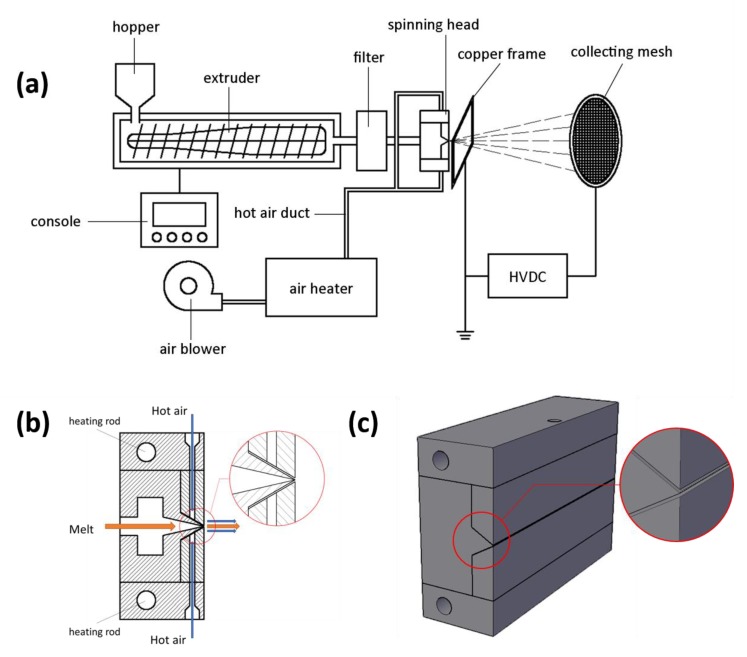
The schematic illustration of the electrostatic-assisted melt-blown system setup (**a**) and the details around the spinning head (**b**,**c**).

The schematic illustration of the electrostatic-assisted melt-blown system setup and the details around the spinning head are shown in Figure 1. The melt-blown equipment (SH-RBJ) was produced by Shanghai Sunhoo Automation Equipment Co., Ltd. (Shanghai, China). It has a hopper which feeds the raw material into the machine, a heated extruder with a rotating screw inside which pushes material forward, a filter which removes impurities from the melt, and a spinning head. The air blower (TF-65) was produced by Kunshan Ta-Fan Blower Co., Ltd. (Kunshan, China). The high velocity air is generated by the blower, heated by the air heater, and then exits from narrow air gaps of the spinning head. The spinning head has a rectangular shape of about 30 cm in length and is made of steel. It is also heated by a pair of heating rods. There are more than 500 orifices distributed in the middle of the spinning head, and the melt exits the spinning head through the orifices and is drawn into filaments at the orifices by hot air from the air passage. Placed adjacent to the spinning head is a grounded copper frame through which the polymer fiber melts are blown. The collecting mesh is connected to a negative high-voltage DC power source (DW-N503-1ACDF, Dongwen, Tianjin, China) to supply a high-voltage electrostatic field between the frame and the mesh. Here, the high-voltage electrostatic field is applied between the copper frame positioned 2 cm away from the MB head and the collecting mesh, where the electric field intensity is simply treated as a rectangular uniform electric field. The distance between the copper frame and collecting mesh can be adjusted. Polypropylene pellets were fed into the hopper and melted in the extruder. The molten polymer was then extruded out of the spinning head and drawn by the high-velocity hot air. Simultaneously, the electrostatic field between the frame and the mesh helped attenuate fiber diameter to form microfibers. Finally, the fabric was formed on the collecting mesh. A continuous process setup has also been designed and will be discussed in a later communication.

### 2.3. Preparation of PP Nonwoven Fabrics

Polypropylene was heated to 265 °C in the extruder and pushed through with a pump rate of 10 g·min^−1^. The temperature of the hot air was 255 °C, and the flow rate of air was 1.7 m^3^·min^−1^ (see in Supplementary Material). The velocity of the air at the exit of nozzle was calculated to be a few thousand meters per minutes, much lower than the sonic speed. When the collecting distance (20 cm) between the copper frame and the collecting mesh was fixed, the voltage (0, 10, 20, 30, and 40 kV) of the DC power was varied to explore the effect of electric field intensity on fiber diameter. When the DC voltage (40 kV) was fixed, the collecting distance (10, 15, and 20 cm) was varied by moving the position of the copper frame to examine the effect of the electric field distance on fiber diameter. The sample was not collected until the machine had run for 0.5 h to produce stable fabric. To ensure consistent fabric thickness, each sample was collected for a continuous production time of 30 s. In addition, the yield of the fabric in this work was 10 g·min^−1^ in the experiment. It was not affected by application of the electric field and was affected only by the extrusion rate of melt blown.

### 2.4. Characterization

The morphology and the structure of the PP nonwoven fabric were characterized by a scanning electron microscope (SEM, Hitachi S-4800, Tokyo, Japan). All samples were plated with a thin layer of platinum before SEM imaging to ensure high electrical conductivity. An image processing software (Nano Measurer version 1.2, Shanghai, China) was used to measure the diameter of the fibers. Four sets of positions were randomly selected for each set of samples and more than 100 fibers were counted to obtain the fiber diameter distribution and the mean of the fiber diameter.

The stress-strain curve of the fabric was measured by the Instron Universal Testing System (Instron 5300 Floor Model Universal Testing System, Norwood, MA, USA). The fabric samples were cut into strips of 5 cm in length and 1 cm in width. Each group included 10 strips and their thicknesses were measured by a fabric thickness meter (YG141A fabric thickness meter, Wenzhou, China). The stretching speed was set to 10 mm/min, and the clamping distance was set to 20 mm.

The filtration efficiency refers to the ratio of the dust of a certain diameter that is filtered out to the concentration of the dust in the aerosol before being filtered when the aerosol passed through the filter material. It was tested by a filter media test system (Topas AFC 131, Dresden, Germany). The probe in the system can separately measure the concentration of dust in the aerosol before and after filtration, hence the filtration efficiency was able to be calculated. Di-ethyl-hexyl-sebacat (DEHS) was used as the test aerosol. The concentration of the aerosol was 1.0 mg·m^−3^ and the flowrate of air was set to 10.0 m^3^·h^−1^. The pore size distribution was measured by a pore size meter (Topas PSM 165, Dresden, Germany). The wetting fluid was Topor (perfluoro compound, Topas specific testing fluid, surface tension 16 mN·m^−1^). The testing cross-sectional area was 0.95 cm^2^. The flow rate range of the compressed air was from 0.06 to 70.00 L·min^−1^, and the maximum pressure was 1000.00 mbar. Each group was measured five times, and the filtration efficiency and the pore size distribution were recorded and averaged from five measurements. The filtration efficiency curve and the pore size distribution diagram were drawn from the above data.

In addition, the air permeability of the fabric was measured by an air permeability tester (Textest FX 3300-IV, Schwerzenbach, Switzerland). The testing pressure was set to 200 Pa, and the measured area was 20 cm^2^. The air permeability of each group was tested based on 10 sets of data, and the mean of the data was taken.

## 3. Results and Discussion

### 3.1. Morphology and Structure

The samples produced under different conditions were observed by SEM, and a series of images of fabric morphology were obtained. Then, an image processing software (Nano Measurer version 1.2, Shanghai, China) was used to measure the fiber diameter.

**Figure 2:**
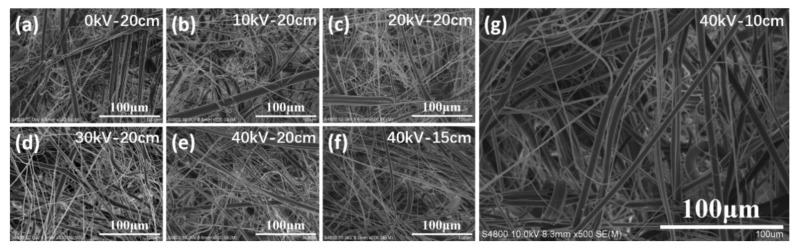
SEM graphs of the polypropylene (PP) microfiber prepared under different voltages or electric field distances by electrostatic-assisted melt blown. (**a**) to (**e**) show PP microfiber prepared in voltage of 0 kV (**a**), 10 kV (**b**), 20 kV (**c**), 30 kV (**d**) and 40 kV (**e**); (**e**) to (**g**) show PP microfiber prepared in electric field distance of 20 cm (**e**), 15 cm (**f**) and 10 cm (**g**).

**Figure 3:**
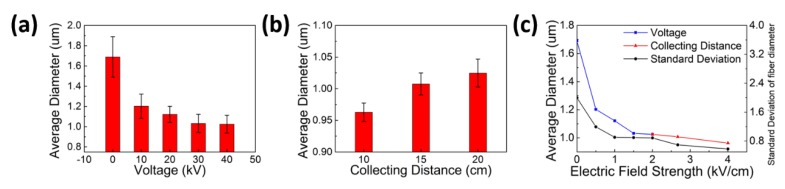
Fiber diameter of fabric prepared under different voltages (**a**), collecting distances (**b**), and electric field strength’s influence on average diameter (**c**). The error bars in (**a**,**b**) represent the standard deviation of fiber diameter for each group of samples.

SEM graphs of the PP microfiber prepared under different voltages are shown in Figure 2a–e, and the fiber diameter analysis of these fabrics is shown in Figure 3a. As shown in these figures, under a fixed electric field distance of 20 cm, as the voltage increased from 0 to 40 kV, the mean of the fiber diameter was reduced from 1.69 to 1.02 μm, and the uniformity of the diameter was improved. SEM graphs of the PP microfiber prepared under different electric field distance are shown in Figure 2e–g, and the diameter analysis of these fabrics is shown in Figure 3b. The voltage of the DC power was set to 40 kV, and the electric distance was decreased from 20 to 10 cm. As is shown in these figures, with the decrease of electric distance, the mean of the fiber diameter decreased from 1.02 to 0.96 μm. Meanwhile, the fiber diameters became more uniform.

As the voltage increased, the electric field distance decreased, which led to an increase in the electric field strength. The relationship between electric field strength and mean of fiber diameter is shown in Figure 3c. The blue line represents that electric field strength was varied by adjusting voltage, and the red line shows that it was changed by adjusting the electric field distance. With the enhancement of the electric field strength, the mean of the fiber diameter decreased. As is shown in the data, increased electric field strength leads to finer fibers and narrower diameter distribution. The influence of the distance between the copper frame and the collecting mesh on the fiber diameter is smaller than that of the voltage on the fiber diameter. Since the distance is reduced by moving the copper frame away from the spinning head, most of the melt away from the spinning head has cooled into filament, and the drafting effect of the electric field is weakened there. However, the drawing effect still has an increasing trend due to the increase of the electric field force.

The introduction of the electrostatic field, especially the enhancement of electric field intensity, can effectively reduce the fiber diameter. Some theoretical work has been reported to support this conclusion [31,32,33,34,35]. In Equation (1) [31], *r_t_* denotes the fiber diameter after a series of unstable motions in the electric field, which was influenced by surface tension *γ*, flow rate ε, current *I*, dielectric constant *ε*, and unstable dimensionless wavelength *ϕ*. The formula is widely regarded as the limiting diameter model for the stretching of a viscous charged fluid in an electric field. Some studies [32,33,34] have given the experimental proofs for the conclusion that the increase of voltage can effectively reduce the diameter of the fiber in melt electrospinning. Wang et al. [35] also talked about the relationship between fiber diameter and uniformity and applied voltage. In this work, the flow rate *ε* is the melt extrusion rate through the MB spinning head, and the current *I* is mainly produced by the polarization charge in the electrostatic field. In this case, the larger the voltage, the greater the current, which further creates a finer fiber diameter. As we can see from Figure 3a,c, the fiber diameter was obviously reduced from 1.69 to 1.02 μm with the increase of voltage on a fixed spinning head to copper frame distances at 20 cm. However, the fiber diameter was reduced only slightly, from 1.02 to 0.96 μm, when the voltage was fixed at 40 KV and the distance was reduced from 20 to 10 cm. In this situation, the electric field strength was doubly increased, but the fiber diameter was not obviously reduced, as shown by the red line in Figure 3c. However, we are still convinced that the increase of electric field strength can effectively reduce the diameter of the fiber, and the best way to increase the electric field strength is by increasing the voltage instead of reducing the spinning distance. Here, the thicker fiber diameter was perhaps caused by the reduction of the spinning distance, thus leaving insufficient space for the further stretching of the jet to form thinner fibers.

(1) $$r_{t}={\left[\gamma \varepsilon \frac{Q^{2}}{I^{2}}\frac{2}{\pi \left(2\ln\phi -3\right)}\right]}^{1/3}$$

**Figure 4:**
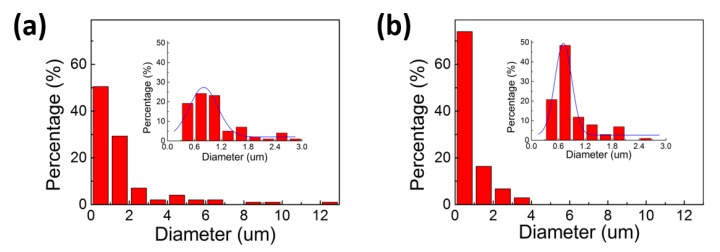
Fiber diameter distributions of PP nonwoven fabric prepared by conventional melt blowing (**a**) and electrostatic-assisted melt blown (**b**). Insets are the diameter distributions of each group in the 0–3 μm range.

Finally, samples with the smallest diameter and the best uniformity were chosen as a follow-up experiment group. The voltage of this group was 40 kV and the electric field distance was 10 cm. Conventional melt-blown fabric was selected as the control group. The diameter analysis of the two groups is shown in Figure 4, and the blue curves represent the general trend of fiber diameter change. According to Figure 4, the electrostatic-assisted melt-blown fabric has more fine fibers, especially at 0.6–0.9 μm, and the fiber diameter distribution of electrostatic-assisted melt-blown fabric is concentrated at 0–4 μm, which leads to a decrease in mean diameter. By introducing the electric field to the melt-blown process, the mean diameter of the fabric is decreased from 1.69 to 0.96 μm, which is about a 40% reduction in fiber diameter.

### 3.2. Stress and Strain

**Figure 5:**
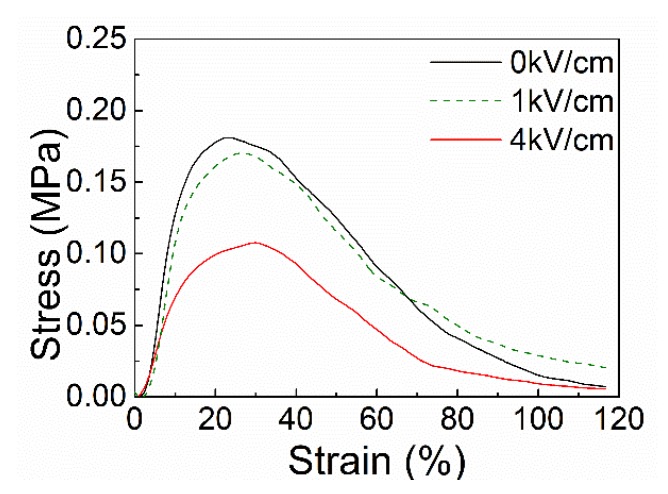
Stress-strain curve of electrostatic-assisted melt-blown fabric and conventional melt-blown fabric.

Stress-strain curves of the experimental group and the control group are shown in Figure 5. It can be drawn from the figure that the maximum strength of electrostatic-assisted melt-blown fabrics is about 40% lower than that of conventional melt-blown fabrics, and it decreases from 0.18 to 0.11 MPa. The Young’s modulus of each group was measured to be 1.77 MPa for 0 kV/cm, 1.72 MPa for 1 kV/cm, and 0.80 MPa for 4 kV/cm. This is due to the decrease of the fiber diameter. Tensile properties of nonwovens are related to their structure. The structure of nonwoven fabric is an irregular net structure, the orientation of the fibers varies, and fibers are bonded together by bonding points. When the nonwoven fabric is stretched, the stress of the fabric is related to the stress of the fiber and the strength of the bonding points [2,36]. The strength of the fabrics becomes weaker as the melt-blown fibers become finer due to the reduction of effective bonding between the fibers. This observed phenomenon may be explained by the higher degree of cooling on the finer fibers when they make contact with each other, which results in reduced bonding effectiveness among the fibers, forming a fabric of weaker strength [2,37].

### 3.3. Pore Size Distribution and Air Permeability

**Figure 6:**
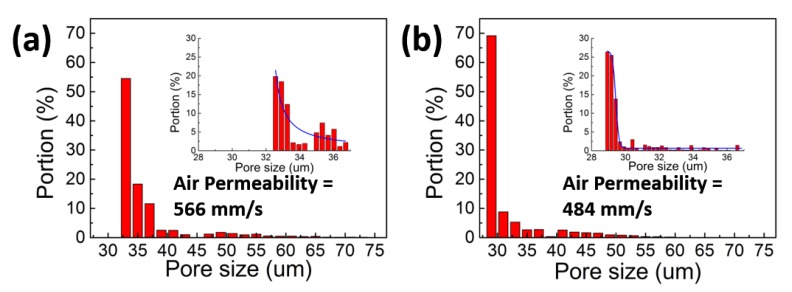
Pore size distribution and air permeability of conventional melt-blown fabric (**a**) and electrostatic-assisted melt-blown fabric (**b**).

The pore size distribution and air permeability of conventional melt-blown fabric and electrostatic-assisted melt-blown fabric are shown in Figure 6. The graph shows that electrostatic-assisted melt-blown fabric has an average pore size of 29.285 μm and conventional melt-blown fabric has an average pore size of 33.415 μm. The mean pore size of electrostatic-assisted melt-blown fabric is slightly smaller than that of melt-blown fabric, and the pore size distribution of electrostatic-assisted melt-blown fabric is more concentrated than that of melt-blown fabric. The electrostatic-assisted melt-blown fabric has a finer fiber diameter due to the auxiliary draft of the electrostatic force. The distribution of fibers within the unit area becomes denser and the fibers become more intertwined. So, the mean pore size of the fabric becomes smaller and the distribution of the fiber diameter becomes symmetrical, which also makes the distribution of the pore size more concentrated. Denser fibers, more intertwined fibers, and smaller pore size lead to lower air permeability [38].

### 3.4. Filtration Efficiency

**Figure 7:**
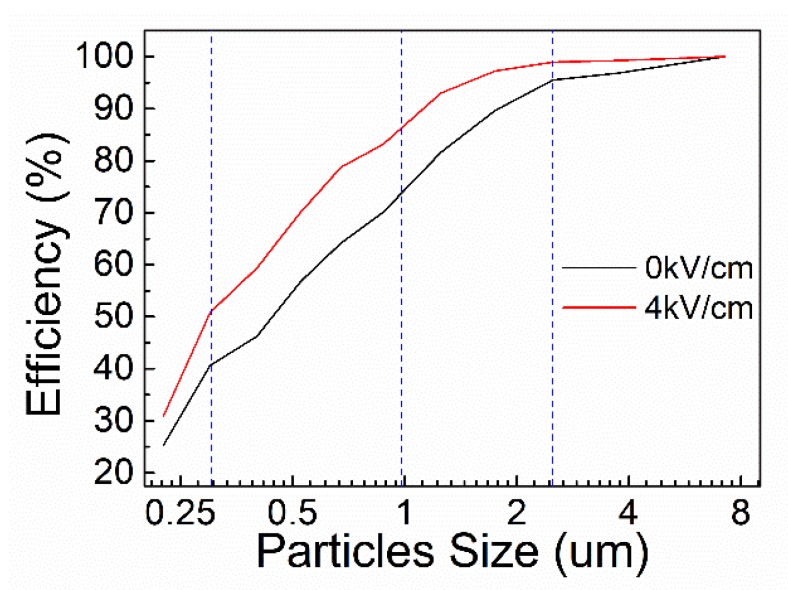
Filtration efficiency of melt-blown fabric and electrostatic-assisted melt-blown fabric.

The filtration efficiency of the two groups is shown in the Figure 7. It can be seen from the figure that the filtration efficiency of the electrostatic-assisted melt-blown fabric is better than that of the melt-blown fabric. The filtration efficiencies of three particle sizes are listed in Table 1 to show that electrostatic-assisted melt-blown fabric has better filtration efficiency than ordinary melt-blown fabric. The main factors affecting fabric filtration efficiency are pore size and fiber diameter. As the fiber diameter decreases, the pore size becomes smaller and the distribution of fibers per unit area is denser. When the aerosol flows, the dispersion of air flow is enhanced by the fabric, giving the particles in the aerosol more chance to adhere to the fabric [39]. Meantime, electrostatic-assisted melt blown causes a small amount of charge in the fabric, which enhances the adsorption capacity of the fabric. This allows the fabric to improve filtration efficiency [40].

## 4. Conclusions

In summary, an electrostatic field was applied directly to the melt-blown spinning head to achieve a combination of melt blown and electrical field effects. This approach is more productive than the electrospinning process. In this way, we produced polypropylene microfibers about 40% finer than conventional melt-blown fiber, with an average diameter of 0.96 and a narrower fiber size distribution. The strength, pore size distribution, and filtration efficiency of conventional melt-blown fabrics and electrostatic-assisted melt-blown fabrics were tested. The results show that electrostatic-assisted melt-blown microfibers have better filtration efficiency, which may be used in air filtration.

## Figures and Tables

**Table 1:** Filtration efficiencies of different particle sizes.

Particle Size	0.3 μm	1 μm	2.5 μm
Melt-blown	40.651%	73.986%	95.353%
Electrostatic-assisted Melt-blown	50.826%	86.442%	98.969%

## Supplementary Materials

The following are available online at http://www.mdpi.com/2073-4360/10/9/959/s1. Figure S1. SEM graphs of the PP fiber prepared by melt blown under different melt temperatures: 220 °C (a), 240 °C (b), 260 °C (c), 280 °C (d), and 300 °C (e). The fiber diameter analysis of these fabrics is shown in (f). Figure S2. SEM graphs of the PP fiber prepared by melt blown under different air temperatures: 220 °C (a), 240 °C (b), 260 °C (c), 280 °C (d), and 300 °C (e). The fiber diameter analysis of these fabrics is shown in (f). Figure S3. SEM graphs of the PP fiber prepared by melt blown under different extruder screw rotating speeds: 2 Hz (120 rpm) (a), 3 Hz (180 rpm) (b), 4 Hz (240 rpm) (c), and 5 Hz (300 rpm) (d). The fiber diameter analysis of these fabrics is shown in (e). Figure S4. SEM graphs of the PP fiber prepared by melt blown under different blower speeds: 15 Hz (90 rpm) (a), 20 Hz (1200 rpm) (b), 25 Hz (1500 rpm) (c), 30 Hz (1800 rpm) (d), and 35 Hz (2100 rpm) (e). The fiber diameter analysis of these fabrics is shown in (f). Figure S5. SEM graphs of the PP fiber prepared by melt blown under different die-to-collector distances (DCDs): 20 cm (a), 25 cm (b), 30 cm (c), 35 cm (d), and 40 cm (e). The fiber diameter analysis of these fabrics is shown in (f).
