# Supplementary material for: Preparation of Polypropylene Micro and Nanofibers by Electrostatic-Assisted Melt Blown and Their Application
Source: Polymers (Basel). 2018 Aug 29;10(9):959. doi: 10.3390/polym10090959 (PMC6403903; doi:10.3390/polym10090959)
Supplement: Supplementary file 1 [file polymers-10-00959-s001.pdf]

Supplementary Material

In order to obtain meltblown fiber with good appearance, factors affecting fiber morphology and nonwoven properties including air temperature, melt temperature, melt throughput (expressed as extruder screw rotating speed), primary airflow rate (expressed as blower speed) and die-to-collector distance (DCD) were explored systematically. The results are shown in Figures 1 to 5. After a series of detailed studies, air temperature was set to 255 °C, melt temperature was set to 265 °C, melt throughput was set to 10 $g \cdot min^{-1}$ (extruder screw rotating speed 60 rpm), primary airflow rate was set to 1.7 $m^3 \cdot min^{-1}$ (blower speed 1800 rpm) and DCD was set to 20 cm.

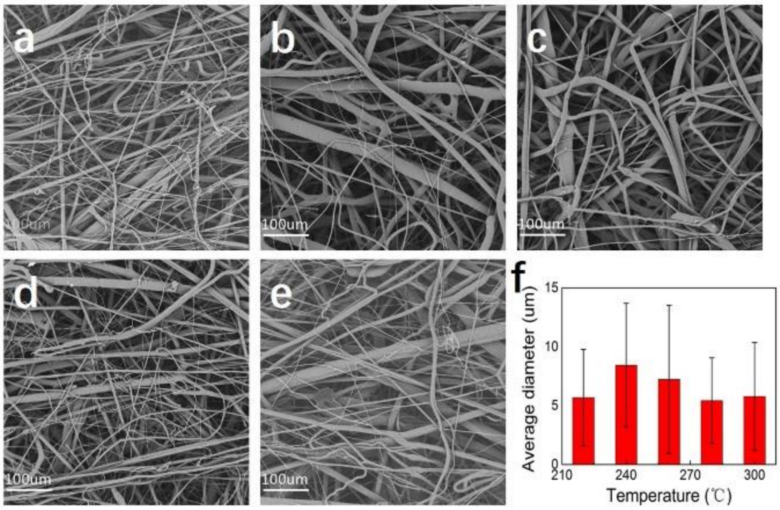

Figure 1 SEM graphs of the PP fiber prepared by meltblown under different melt temperatures. 220°C (a), 240°C (b), 260°C (c), 280°C (d), 300°C (e). And the fiber diameter analysis of these fabrics (f).

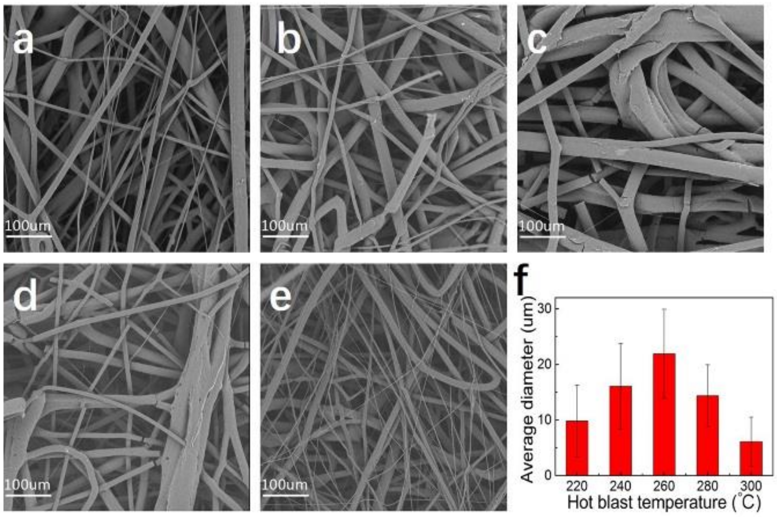

Figure 2 SEM graphs of the PP fiber prepared by meltblown under different air temperatures. 220°C (a), 240°C (b), 260°C (c), 280°C (d), 300°C (e). And the fiber diameter analysis of these fabrics (f).

According to Figures 1 and 2, the effect of temperature on fiber fineness is not significant, but it affects the quality of the fabric. When the temperature is too low or too high, non-smooth particles (melt didn't drawn into fibers by hot air) appear on the surface of the fabric, affecting the quality of the product.

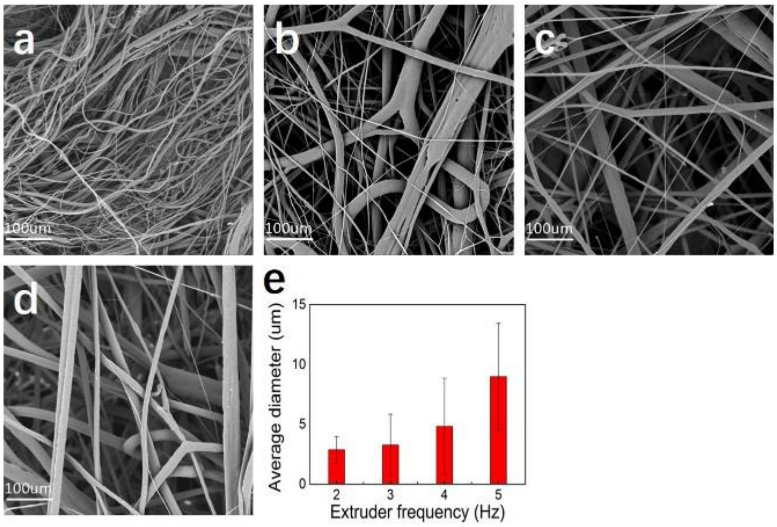

Figure 3 SEM graphs of the PP fiber prepared by meltblown under different extruder screw rotating speeds. 2 Hz (120 rpm) (a), 3Hz (180 rpm) (b), 4 Hz (240 rpm) (c), 5 Hz (300 rpm) (d). And the fiber diameter analysis of these fabrics (e).

As is shown in Figure 3, melt throughput has the greatest influence on fiber fineness. The lower melt throughput, the more adequate the hot air drafts the melt thus benefiting the formation of finer fibers. Nevertheless, fiber diameter would not be further reduced at a too low melt throughput due to the necking phenomenon. On the other hand, at an over high melt throughput, the melt has not been fully stretched before it is collected at the mesh.

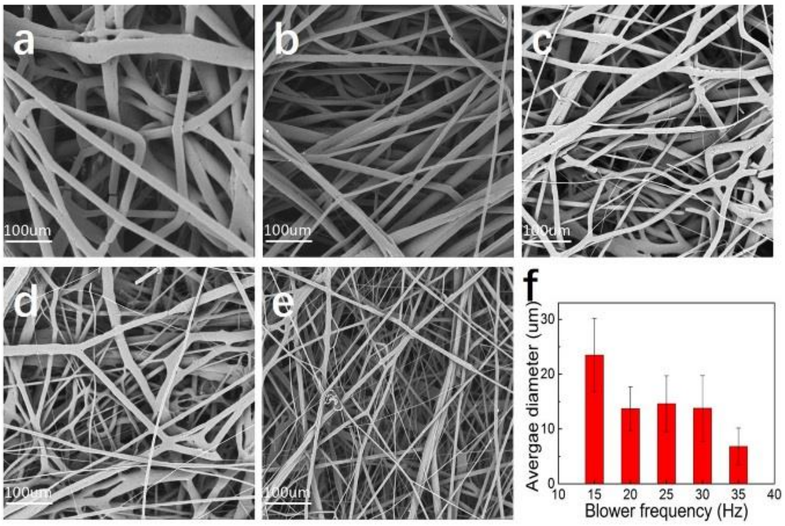

Figure 4 SEM graphs of the PP fiber prepared by meltblown under different blower speeds. 15 Hz (90 rpm) (a), 20 Hz (1200 rpm) (b), 25 Hz (1500 rpm) (c), 30 Hz (1800 rpm) (d), 35 Hz (2100 rpm) (e). And the fiber diameter analysis of these fabrics (f).

According to Figure 4, primary airflow rate has a great influence on fiber fineness. As airflow rate increases, the diameter of the fiber decreases. Primary airflow rate has also a great impact on fabric strength. Higher airflow rate improves fabric strength due to higher single fiber strength and better thermal bonding.

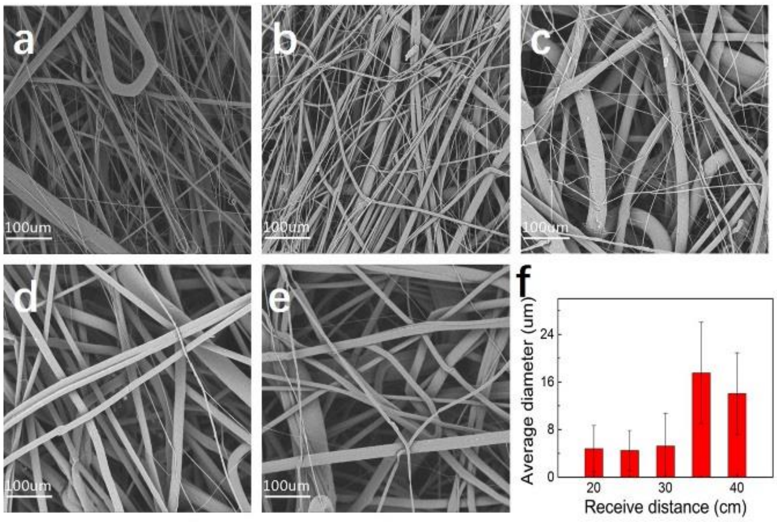

Figure 5 SEM graphs of the PP fiber prepared by meltblown under different DCDs. 20 cm (a), 25 cm (b), 30 cm (c), 35 cm (d), 40 cm (e). And the fiber diameter analysis of these fabrics (f).

As is shown in Figure 5, DCD has a great influence on fiber fineness. At higher DCD, the fiber web becomes bulky and breathable. However, when DCD is too low, the melt has not been fully stretched before it reaches the coagulation drum and thus is difficult to form filaments.
